# Functional Characterization of Melanocortin-3 Receptor in a Hibernating Cavefish *Onychostoma macrolepis*

**DOI:** 10.3390/ani12010038

**Published:** 2021-12-25

**Authors:** Lian Wu, Huixia Yu, Haolin Mo, Xianyong Lan, Chuanying Pan, Lixin Wang, Haiyu Zhao, Jishu Zhou, Yang Li

**Affiliations:** 1 College of Animal Science and Technology, Northwest A&F University, Xianyang 712100, China; lxw784@alumni.bham.ac.uk (L.W.); yuhuixia@nwsuaf.edu.cn (H.Y.); 2017010832king@nwsuaf.edu.cn (H.M.); lanxianyong79@nwsuaf.edu.cn (X.L.); fisherwanglx@nwsuaf.edu.cn (L.W.); zhoujishu@163.com (J.Z.); 2 School of Life Sciences, Lanzhou University, Lanzhou 730000, China; zhaohy@lzu.edu.cn

**Keywords:** appetite regulation, ligand preference, cAMP signaling, MAPK/ERK pathway, starvation

## Abstract

**Simple Summary:**

In this study we isolated and characterized a gene called *omMc3r* from a hibernating cavefish *Onychostoma macrolepis*. This gene was confirmed by our study to be involved in the regulation of signal pathways related to energy balance and food efficiency. These results can provide clues for exploring the adaptive mechanisms of fish, especially cavefish, with respect to nutrient-poor conditions.

**Abstract:**

Melanocortin-3 receptor (MC3R) plays an important role in the energy homeostasis of animals under different nutritional conditions. *Onychostoma macrolepis* is a hibernating cavefish found in the northern part of the Yangtze River, and its adaptation to a nutrient-poor environment has attracted growing interest. In this study, we characterized the protein structure of *Onychostoma macrolepis* Mc3r (omMc3r), examined its tissue distribution, and investigated its function in mediating cellular signaling. We showed that the CDS of omMc3r is 978 bp, encoding a putative protein of 325 amino acids. Homology and phylogenetic analyses indicated that omMc3r is evolutionary close to cyprinids. Real-time quantitative PCR (RT-qPCR) revealed that omMc3r was highly expressed in the liver and brain. The functions of omMc3r to mediate ligands activating downstream signaling have also been confirmed by using signal pathway-specific reporters. The four agonists α-MSH, β-MSH, NDP-MSH, and ACTH (1–24) can all activate the cAMP and MAPK/ERK signaling pathway, albeit with different potency orders. The “primitive” ligand ACTH (1–24) had the highest potency on the cAMP signaling pathway, while the synthetic ligand NDP-MSH had the highest activation effect on the MAPK/ERK signaling pathway. This research will lay the foundation for studying the energy regulation mechanism of cavefish in an oligotrophic environment.

## 1. Introduction

G protein-coupled receptors (GPCRs) are seven-transmembrane domains (7TMDS), proteins that triggers intracellular signals by coupling with G proteins and arrestins to transmit a large number of extracellular signals. Melanocortin receptors (MCRs) belong to the class A subfamily of G protein-coupled receptors (GPCRs), which has been proved to exist in five members (MC1R-MC5R) in mammals and play versatile roles in physiological processes [1]. Although these five receptors are similar in structure, their tissue distributions and respective cellular functions are quite different. MC1R is mainly expressed in melanocytes and affects skin and hair pigmentation [2]. MC2R is expressed in the adrenal cortex and adipose tissue and regulates steroid synthesis [3]. MC5R is expressed in various peripheral tissues and participates in regulating the function of exocrine glands [4,5]. MC3R and MC4R are mainly expressed in the hypothalamus and are involved in maintaining energy homeostasis [6,7]. The latter two have similar physiological functions; for example, the knockout or loss-of-function mutations of MC3R or MC4R can cause obesity in mice and humans [8,9]. However, the molecular mechanisms that cause this phenotype are not the same. MC3R regulates energy homeostasis through food efficiency and circadian rhythms [10], while MC4R mainly affects food intake and energy expenditure [11].

The endogenous agonists of MC3R are the cleavage products of pro-opiomelanocortin (POMC), including α-/β-/γ-melanocyte-stimulating hormone (MSH) and adrenocorticotropin (ACTH) [12]. Prior studies have found that MC3Rs in human and fish show different selectivity for ligands. Human MC3R has a unique preference for γ-MSH [3], while the Mc3r (according to the fish protein nomenclature, the MC3R in fish should be written as Mc3r) of spiny dogfish [3] and channel catfish [12] have the highest affinity for ACTH. With regard to intracellular signal transduction, MC3R can couple to Gαs and Gαi subunits to elevate cyclic adenosine monophosphate (cAMP) and activate the MAPK/ERK signaling pathway, respectively [13,14].

The lack of Mc3r in some teleosts, such as fugu [15], medaka [16], and stickleback [17], restricts the cloning and functional studies of this gene in fish. To date, only the MC3Rs from blunt snout bream [18], zebrafish [16,19], spiny dogfish [3], stingray [20], channel catfish [12], and topmouth culter [21] have been cloned and the latter five had their pharmacological functions demonstrated.

*Onychostoma macrolepis* is a cavefish belonging to the family Cyprinidae. These fish lives in mountain streams at an altitude of 270–1500 m and feed on aquatic insects and algae. In addition, *O. macrolepis* enters the cave to hibernate from November to April of the following year, and, so far, only hibernating fish have been found in the northern part of the Yangtze River [22]. Given that the mutation of *Mc4r* is critical for the survival of Mexican cavefish (*Astyanax mexicanus*) in starvation [23], cloning and pharmacological studies of the *Mc3r* of *O. macrolepis* may provide important clues for revealing the mechanisms that have evolved to adapt cavefish to oligotrophic environments. In this study, we cloned the coding sequences (CDS) of *Mc3r* from *O. macrolepise*, examined its tissue distribution, and demonstrated its pharmacological activation with treatments of four agonists.

## 2. Materials and Methods

### 2.1. Chemicals, Reagents, and Primers

The four agonists (α-MSH, β-MSH, [Nle^4^, D-Phe^7^]-α-MSH (NDP-MSH), ACTH (1–24)) were purchased from GenScript Biotechnology (Nanjing, China). The NCBI online tool (https://www.ncbi.nlm.nih.gov/tools/primer-blast/, accessed on 12 April 2021) was used to design conventional PCR and real-time quantitative PCR primer pairs. The primer sequences are shown in Table 1 and were synthesized by Sangong Biotech (Shanghai, China). Two restriction enzymes, *EcoR*I and *BamH*I, were purchased from TaKaRa Biotechnology (Dalian, China).

### 2.2. Total RNA Extraction and Reverse Transcription (RT)

The *O. macrolepis* used in this experiment were obtained from the Ankang Fisheries Experimental and Demonstration Station, Northwest A&F University (Shaanxi, China). A total of nine fish were used in this study, with a mean (SD) body length of 23.5 ± 3 cm. The fish were euthanized with 2-phenoxyethanol at a dilution of 1:500 and dissected quickly. The brain, gonads, liver, heart, intestines, spleen, kidneys, and muscle were collected for RNA extraction. Total RNA of each tissue was extracted using the Trizol method. The quality of total RNA was determined by 260/280 ratios with a NanoDrop ND-1000 spectrophotometer (NanoDrop Technologies, Wilmington, DE, USA). The samples with a 260/280 ≥ 1.9 were used for cDNA synthesis and they were treated with a Turbo DNase kit (Ambion, Austin, TX, USA) before use.

To begin the reverse transcription process, 5 μg total RNA and 1 μL olio-deoxythymidine were premixed with a total volume of 12 μL, incubated at 70 °C for 4 min, and immediately cooled on ice for 2 min. Then, 5× reaction buffer, 20 mM deoxynucleotide triphosphate (dNTP), and 200 U Moloney murine leukemia virus reverse transcriptase (M-MLV) were added into the mix to a total volume of 20 μL. The reverse transcription reaction was performed at 42 °C for 1 h, followed with 70 °C heating for 5 min to inactivate the reaction. These products were stored at −20 °C and were used for subsequent molecular cloning and tissue quantitative expression studies of *omMc3r*.

### 2.3. Molecular Cloning of omMc3r and Construction of Its Eukaryotic Expression Plasmid

The CDS of *omMc3r* was obtained from our transcriptome library. The transcriptome library was constructed and sequenced by Biomarker Technologies (Beijing, China). Specifically, the transcriptome library was generated using a NEBNext^®^ Ultra^TM^ RNA Library Prep Kit (NEB, Carlsbad, CA, USA), then purified by the AMPure XP system (Beckman Coulter, Beverly, MA, USA). Complementary DNA (cDNA) fragments of 250–300 bp were selected and their quality was assessed by the Agilent Bioanalyzer 2100 system (Agilent Technologies, CA, USA). The qualified library was sequenced by Illumina HiSeq 2000 to obtain 150 bp paired-end reads. Clean reads were obtained by removing reads with sequencing adapters, unknown nucleotides (N ratio > 10%), and low quality (quality score < 20). The zebrafish genome (GRCz11) was used as a reference for subsequent de novo assembly and annotation.

To verify the annotated *omMc3r* sequence in the transcriptome library, we used a cDNA pool mixed with cDNA from various tissues as a template to carry out PCR amplification. The reaction was performed in a 10 μL reaction system, including 1 μL of cDNA template, 5 μL of 2 × Taq PCR Master Mix (Dye) (Biosune, Shanghai, China), and 0.2 μM upstream and downstream primers. The PCR procedure was as follows: initial denaturation for 4 min at 95 °C; 35 cycles of 95 °C for 30 s, 59 °C for 30 s, and 72 °C for 1 min; 72 °C for 10 min to fully extend the amplicons. The amplified products were subjected to 1.5% agarose gel electrophoresis to visualize band size. The PCR fragments with expected size were purified by a DNA gel extraction kit (Tiangen, Beijing, China), and then sub-cloned to a pGEM-T easy vector (Madison, WI, USA). The recombinant plasmids were transformed into *Escherichia coli* (*E. coli*) DH5α to select the positive clones. The selected clones were sequenced by Sangong Biotech (Shanghai, China).

In order to test whether omMc3r can be activated by ligands in vitro, we sub-cloned the coding region of *omMc3r* into pcDNA3.1 (+) vector (Invitrogen, Carlsbad, CA, USA) for subsequent transfection of cells. The constructed vector was further verified by sequencing before use.

### 2.4. Homology and Phylogenetic Analyses of omMc3r

Multiple alignments based on MC3R amino acid sequences of different species were carried out with DNAMAN 9.0 software (Lynnon Biosoft, CA, USA). The calculations of similarity and alignment annotation were made with the software Gendoc (http://www.nrbsc.org/downloads/, accessed on 14 April 2021). The putative transmembrane domains (TMDs) of omMc3r were predicted by the online website NCBI Conserved Domain Search (https://www.ncbi.nlm.nih.gov/Structure/cdd/wrpsb.cgi?, accessed on 14 April 2021). A phylogenetic tree of amino acids was constructed using MEGA X [24] with the neighbor-joining (NJ) method [25]. Bootstrap values were estimated based on 1000 replicates. GenBank accession numbers of the sequences used in the analyses are presented in a Supplementary Table (Table S1).

### 2.5. Tissue Expression of omMc3r

The relative expression level of *omMc3r* in various tissues of the *O. macrolepis* were evaluated using real-time quantitative PCR (qPCR). Two housekeeping genes, *β-actin* and *gapdh* (glyceraldehyde-3-phosphate dehydrogenase), were set as the internal control [26]. Table 1 shows the used primers. The Eco 48 Real Time PCR System (PCRmax Limited, Staffordshire, UK) was chosen to perform the qPCR in a reaction volume of 10 μL, which included 4 μM forward and reverse primers, 5 μL 2 × SYBR Green Mix and 0.5 μL dilute cDNA (200 ng/μL). The following parameters were used for the amplification reaction: incubate at 95 °C for 30 s, then at 94 °C (30 s), 55 °C (30 s), 72 °C (30 s) for 40 cycles. At the end of program, the PCR melting curve was able to be depicted. The 2^−ΔΔCT^ method was used to analyze the relative expression level. Nine fish were used for biological replicates, and qPCR for each sample was performed for six technical replicates. The data were presented as mean ± SEM.

### 2.6. Cell Culture

Human embryonic kidney (HEK293T) cells were cultured in Dulbecco modified Eagle’s medium (DMEM, Invitrogen, Carlsbad, CA, USA) supplemented with 10% fetal bovine serum (FBS, Biological Industries, Kibbutz Beit Haemek, Israel) and placed in a humid environment containing 5% CO_2_ at 37 °C. Cells were passaged every 2 days.

### 2.7. Luciferase Reporter Assay

This study employed the luciferase reporter system to examine whether the four ligands can activate downstream signals through omMc3r. The luciferase reporter vector pGL4.29 and pGL4.33 of Promega (Madison, WI, USA) contain cAMP respose element (CRE) and serum response element (SRE) in their promoter regions, which can monitor the activation of cAMP and MAPK/ERK signaling pathways, respectively. The brief operating procedure was as follows: the HEK293T cells were passaged to a 6-well plate 24 h before transfection. Then, a mixture containing 1000 ng luciferase reporter vector, 500 ng omMc3r expression plasmid (or empty pcDNA3.1 plasmid), 300 ng pEGFP-N1 (as the internal control for transfection normalization), and 4 μL PEI transfection reagent (Fusheng Biotechnology, Shanghai, China) was used to transfect these cells. The transfected cells continued to grow in the original medium for 24 h, then they were pipetted down and transferred to a 48-well plate to grow for another 24 h to reach a density of 2 × 10^5^ cells per well. The four ligands (α-MSH, β-MSH, NDP-MSH, ACTH (1–24)) were diluted to working concentration in serum-free medium, and then added to the 48-well plate to treat cells for 6 h. After processing, the cells were lysed with 1 × passive lysis buffer (Beyotime Biotechnology, Shanghai, China), and the luciferase substrate was added for reaction. An Infinite F200 microplate reader (Tecan, Männedorf, Switzerland) was used to measure the luciferase activities. For each assay, two additional 48-well plates (*n* = 3) were used as technical replicates and data were shown as mean ± SEM.

### 2.8. Statistical Analysis

The 2^−ΔΔCT^ method [27] was employed for the calculation of the expression level of omMc3r relative to the internal reference gene; the one-way ANOVA tests followed by Tukey’s HSD test for post comparison were then used to assess data significance, and *p* < 0.05 was regarded as significant.

The luciferase activities were converted into a change fold of the treatment group relative to the control group (DMEM serum-free medium) and fitted to the dose-response curve based on nonlinear regression analyses.

## 3. Results

### 3.1. Nucleotide and Deduced Amio Acid Sequences of omMc3r

**Figure 1:**
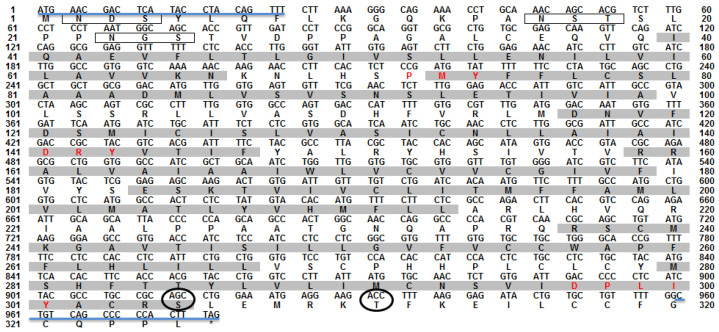
Nucleotide and deduced amino acid sequence of omMc3r. The positions of nucleotides and amino acids are marked on both sides. Gray shading indicates the seven-transmembrane domain (7TMD). Red letters highlight the conserved motifs (PMY, DRY, and DPLIY). Solid black boxes frame the N-glycosylation sites. Black circles represent the phosphorylation sites at the C-terminus. Asterisk (*) denotes a stop codon. The primer pairs utilized for amplification are shown by the blue line.

**Figure 2:**
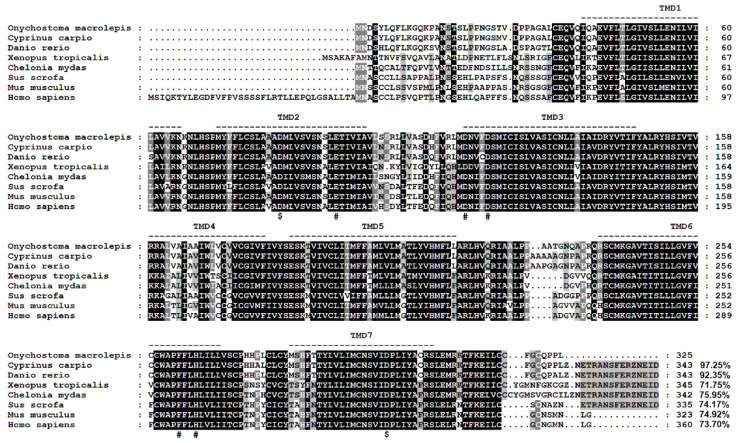
Multiple amino acid sequence alignments of MC3Rs between *O. macrolepis* and other species. Dashed lines represent the transmembrane domains. $ indicates amino acid residues important for ligand selection. # denotes amino acid residues crucial for ligand binding and signaling transduction.

The obtained *omMc3r* (NCBI accession number: MW884251) contains a 978 bp open reading frame, encoding a putative protein of 325 amino acids (Figure 1). The receptor possesses a typical GPCR structure, the 7TMDs. It also has the conserved motifs in MC3Rs, the PMY, DRY, and DPLIY motif, which are located in TMD2, TMD3, and TMD7, respectively. In addition, there are three glycosylation sites at the N-terminus and two phosphorylation sites at the C-terminus (Figure 1). Moreover, residues crucial for ligand binding and signaling in human MC3R (hMC3R) were also found in omMc3r. To be specific, D84 and D297 (D121 and D332 in hMC3R) affect ligand selection, and the residues E94, D117, D121, F260, H263 (D131, D154, D158, F295, H298 in hMC3R) are important for ligand binding and signaling transduction (Figure 2).

Multiple alignments showed that the amino acid sequence of omMc3r was highly conserved in TMDs and intracellular loops (ICLs) but less conserved at the N-terminus and extracellular loops (ECLs) (Figure 2). Similarity calculations indicated that omMc3r has the highest homology with cyprinids (e.g., *Cyprinus carpio* XP_018922723.1: 97.25%, *Danio rerio* AAI62747.1: 92.35%,) and has relatively low homology with mammals (e.g., *Homo sapiens* AAH69105.1: 73.70%, *Sus scrofa* NP_001116609.1: 74.17%).

### 3.2. Phylogenetic Analysis

**Figure 3:**
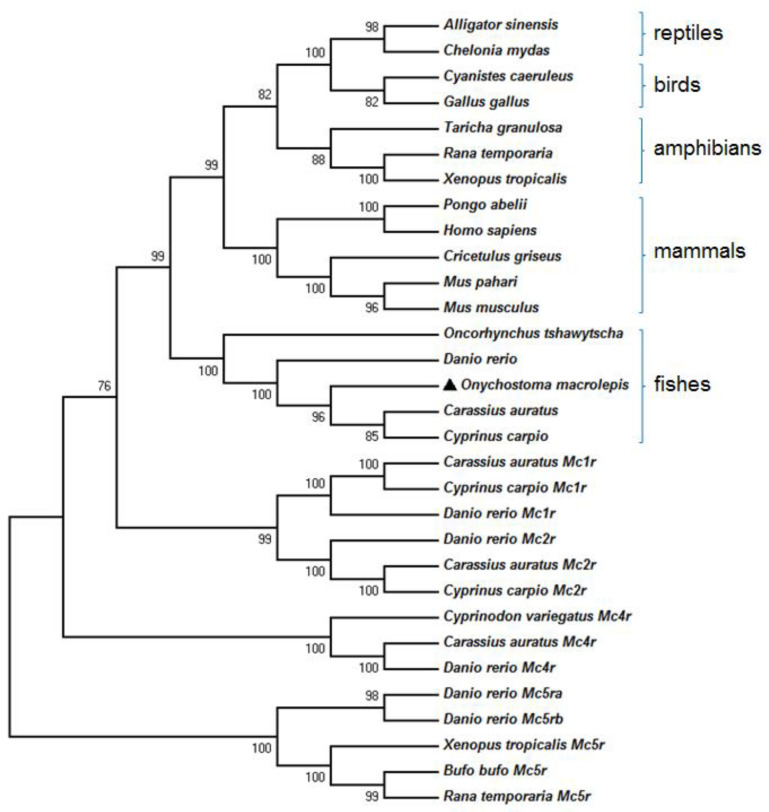
Phylogenetic tree of MC3Rs constructed by the neighbor-joining (NJ) method with MEGA X. The numbers under the nodes represent the bootstrap percentage values from 1000 replicates. The non-Mc3r paralogs together serve as the outgroups. The black triangle indicates omMc3r.

Phylogenetic analysis based on the amino acid sequence showed that the MC3Rs of fish, amphibians, birds, and mammals clustered into one group. The putative protein of omMc3r is classified into the group of bony fish and shows the most recent evolutionary relationship with *Carassius auratus* and *Cyprinus carpio* (Figure 3).

### 3.3. Tissue Expression of omMc3r

**Figure 4:**
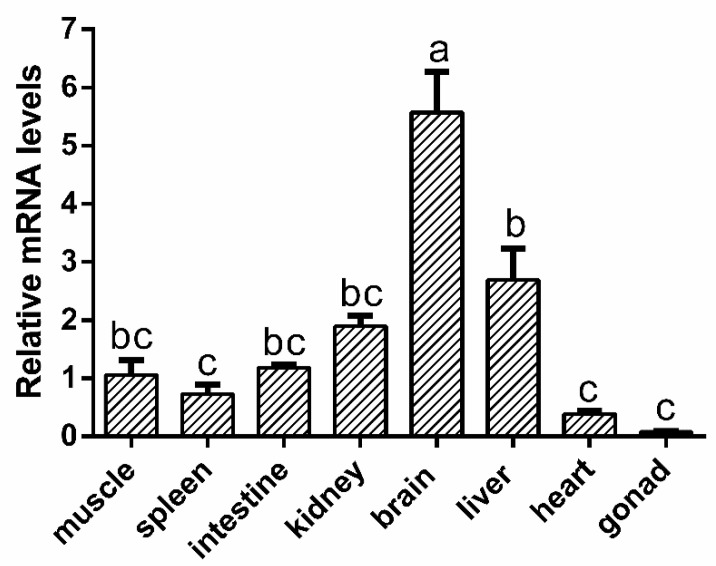
Relative mRNA expressions of *omMc3r* in various tissues. Two housekeeping genes, *β-actin* and *gapdh*, were used as the internal controls. The mRNA level of each gene was normalized to the geometric mean of the expression levels of the internal controls and then calculated as the fold change compared to that of the muscle. The same lowercase letters show no significant difference, while different letters denote significant differences. The test level was 0.05. Data are presented as mean ± SEM (*n* = 9).

Taking the geometric mean of *β-actin* and *gapdh* expression levels as the internal reference, the normalized tissue expression profile showed that the expression of *omMC3R* mRNA was highest in the brain, followed by the liver, kidneys, and muscle. This gene had relatively low expression levels in the heart and gonads (Figure 4).

### 3.4. Functional Characteristics of omMc3r in HEK293T Cells

**Figure 5:**
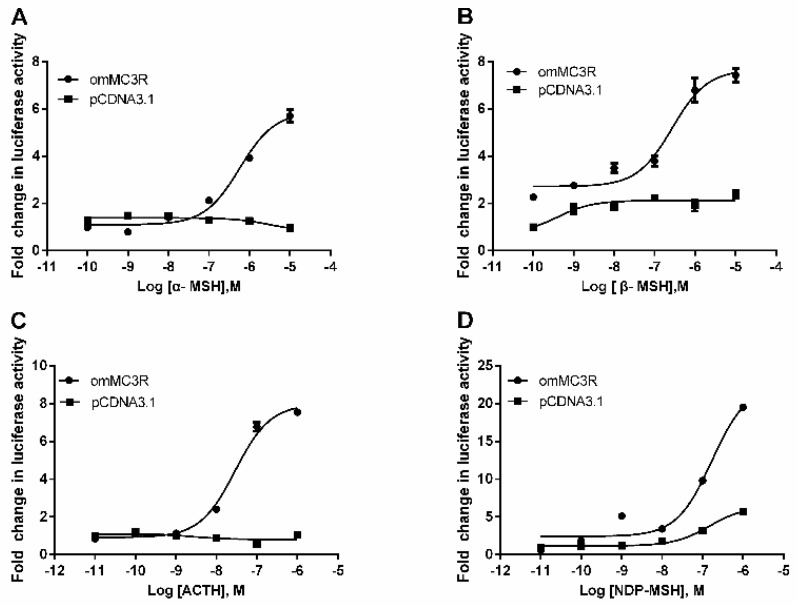
Activation of cAMP signaling mediated by omMc3r. The pGL4-CRE luciferase reporter system (pGL4.29 vector) was used to monitor the effects of α-MSH (**A**), β-MSH (**B**), ACTH (1–24) (**C**), and NDP-MSH (**D**) on the HEK293T cells transiently transfected with omMc3r expression plasmid, as described in Section 2. The results were presented as fold change of the agonist-treated groups relative to the DMEM serum-free medium-treated group. The empty vector pcDNA 3.1 was used as a negative control. Each data point represents mean (SD) of three independent experiments.

**Figure 6:**
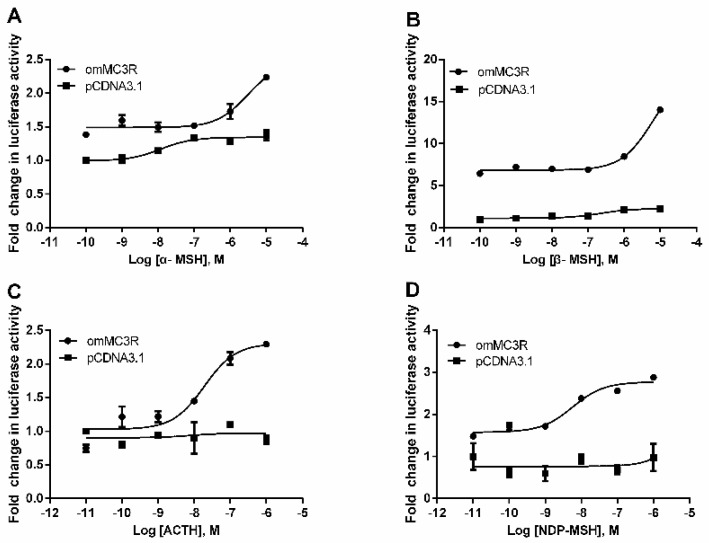
Activation of MAPK/ERK signaling mediated by omMc3r. The pGL4-SRE luciferase reporter system (pGL4.33 vector) was used to monitor the ERK1/2 signaling. HEK293T cells were co-transfected with pGL4.33 and omMc3r vectors were treated with α-MSH (**A**), β-MSH (**B**), ACTH (1–24) (**C**), and NDP-MSH (**D**) for 6 h, and then lysed for measurement of luciferase activity. The results were shown as fold change of the agonist-treated groups relative to the DMEM serum-free medium-treated group, and the empty vector pcDNA 3.1 was used as a negative control. Each data point represents mean (SD) of three independent experiments.

We employed the luciferase reporter systems to investigate the activation properties of omMc3r. Plasmids with CRE (pGL4.29) and SRE (pGL4.33) in the promoter were used to monitor the cAMP and MAPK signaling pathway, respectively. The results, as shown in Figure 5 and Figure 6, indicated that all four agonists, α-MSH, β-MSH, NDP-MSH, ACTH (1–24) can activate cAMP and MAPK/ERK signaling pathways in a dose-dependent manner. However, the potencies of the ligands on these two pathways are not the same (Table 2). For the cAMP signaling pathway, ACTH (1–24) had the highest potency (EC_50_ = 0.029 μM), followed by NDP-MSH (EC_50_ = 0.15 μM), β-MSH (EC_50_ = 0.27 μM), and α-MSH (EC50 = 0.57 μM). As for the MAPK/ERK pathway, the order of potency was: NDP-MSH (EC_50_ = 0.0057 μM) > ACTH (EC_50_ = 0.019 μM) > α-MSH (EC_50_ = 3.06 μM) > β-MSH (EC_50_ = 4.85 μM).

## 4. Discussion

MC3R plays an indispensable role in the regulation of energy homeostasis by controlling feeding efficiency and circadian rhythm [10]. To date, researches on MC3R have mainly focused on mammals; only six studies of Mc3r in fish have been reported, namely, blunt snout bream [18], zebrafish [16,19], spiny dogfish [3], stingray [20], channel catfish [12], and topmouth culter [21]. Given that Mc4r affects the weight loss and fat accumulation of Mexican cavefish in starvation [23], Mc3r, which functions similarly to Mc4r, is also speculated to play an important role in adapting fish to oligotrophic environments. *O. macrolepis* is the only hibernating fish found so far in the northern part of the Yangtze River in China [22]. They live in caves for six months each year. Studying the Mc3r of this species will be helpful in elucidating the mechanism of cavefish adapting to the nutrient-poor conditions. In the present study, we cloned the CDS region of omMc3r, determined its tissue distribution and investigated the activation characteristics of downstream signaling pathways by using luciferase reporter system.

The cDNA we cloned was true *omMc3r*, which was confirmed by the multiple sequence alignment and MC3R-specific structures. Sequence alignment showed that omMc3r was highly conserved in TMDs and ICLs, and it had higher homology with those of crucian carp (*Carassius auratus*), zebrafish (*Danio rerio*), and common carp (*Cyprinus carpio*), and relatively lower homology with those of mammals. A similar conclusion was reached by phylogenetic analyses based on the NJ method. Structurally, omMc3r has some common features of MC3Rs among various species, such as 7TMD and highly conserved PMY, DRY, and DPLIY motifs [28,29]. DPLIY, as one type of NPxxY motif, exists in humans (*Homo sapiens*), mice (*Mus musculus*), western claw frog (*Xenopus tropicalis*), and zebrafish (*Danio rerio*). Interestingly, in rainbow trout and Chinook salmon the NPxxY motif is DPVIY. This indicates that, compared to salmonids, omMc3r is more conserved in evolution. Additionally, in omMc3r we found that the residues important for ligand selectivity and downstream signal transduction are identical to those of human MC3R [30], suggesting that the highly conserved amino acids might constitute the binding pocket for ligands.

MC3R (Mc3r) is known to be mainly expressed in the brain in mammals [1], spiny dogfish [3], and stingray [20]. In our study, real-time qPCR showed that *omMc3r* was predominantly expressed in the brain, and then in the liver, which is consistent with the studies aforementioned. It is worth noting that a recent study in mice has shown that hepatic MC3R is essential for global energy homeostasis and body composition [31]. This study and our results suggest that, in addition to the brain, the liver might be another important organ for Mc3r to regulate global energy metabolism of *O. macrolepis* under nutrient-poor conditions.

MC3R can couple to Gαs and/or Gαi protein to activate downstream signaling pathways [14,32]. Luciferase reporter assay showed that three endogenous ligands (α-MSH, β-MSH, and ACTH (1–24)) and one synthesized ligand (NDP-MSH) could potently activate the Gαs-cAMP and MAPK/ERK signaling pathway through omMc3r. Interestingly, for the above two signaling pathways, the activation potency orders of the four agonists are not the same. For the cAMP signaling pathway, ACTH (1–24) showed the highest activation efficacy, which is consistent with the results of channel catfish [12] but different from those of pigs [33]. ACTH (1–24), as a “primitive” ligand, shows lower and higher affinity to mammals [34] and fish [3,35], respectively. There is a view that ACTH is the “original” ligand for ancestral MCRs [36,37], and our results supported this hypothesis. The MAPK/ERK signaling pathway is involved in the regulation of energy homeostasis [13]. The results for ligand activation showed that the synthetic ligand NDP-MSH has the highest activation potency, which is consistent with the results of studies in mammals [33]. The different activation potency of the original and synthetic ligands on the downstream signaling pathway of omMc3r suggests that the differences in the structure of fish and mammalian MC3R may cause differences in spatial conformations, which in turn affects signaling transduction.

Some limitations in this study are worth noting. Although various GPCR studies from spiny dogfish [3] to channel catfish [12] have used HEK293 cells as a platform, a human-derived cell line may not fully reflect the signal transduction properties of omMc3r. Future research will try to use primary cells isolated from fish to explore the functions of GPCRs.

## 5. Conclusions

In conclusion, we cloned the CDS of Mc3r from *O. macrolepis*, which is evolutionarily conserved and highly expressed in the brain and liver. Functional studies revealed that the agonists α-MSH, β-MSH, NDP-MSH, and ACTH (1–24) could activate cAMP and the MAPK/ERK signaling through omMc3r with different degrees of potency. This research lays the foundation for studying energy regulation in cavefish under conditions of nutrient deficiency.

## Figures and Tables

**Table 1:** Primers for molecular cloning and real-time quantitative PCR.

Primer Name	Primer Sequence	Product Size	Ta ^1^
omMc3r-F	ATGAACGACTCATACCTACAGTTT	978 bp	58 °C
omMc3r-R	CTAAAGTGGGGGCTGACAG		
qPCR-F	CTTCTCGCCAGACTTCACG	100 bp	55 °C
qPCR-R	TCACGGCTCCCTTCATACA		
β-actin-F	TCCACCCGCGAGTACAACCTT	173 bp	57 °C
β-actin-R	CCCACGATGGAGGGGAAGAC		
gapdh-F	TGTGGGCAAAGTCATTCCTG	139 bp	54 °C
gapdh-R	GACAGACTCCTTGATGTTAGCGTA		

^1^ Ta, annealing temperature.

**Table 2:** The signaling properties of omMc3r in response to ligand stimulation.

Ligand	cAMP Response	ERK1/2 Response
	EC_50_ (μM)	EC_50_ (μM)
α-MSH	0.57 ± 0.00478	3.06 ± 0.98
β-MSH	0.27 ± 0.00495	4.85 ± 0.0107
ACTH (1–24)	0.029 ± 0.00706	0.019 ± 0.00128
NDP-MSH	0.15 ± 0.00197	0.0057 ± 0.0012

## Data Availability

All data are included in the article.

## Supplementary Materials

The following supporting information can be downloaded at: https://www.mdpi.com/article/10.3390/ani12010038/s1, Table S1: GenBank accession numbers of amino acid sequences used in multiple sequence alignment and phylogenetic analysis.
