# Supplementary material for: Functional Characterization of Melanocortin-3 Receptor in a Hibernating Cavefish Onychostoma macrolepis
Source: Animals (Basel). 2021 Dec 25;12(1):38. doi: 10.3390/ani12010038 (PMC8749556; doi:10.3390/ani12010038)
Supplement: Supplementary file 1 [file animals-12-00038-s001.zip › animals-1430599-supplementary.pdf]

Table S1. GenBank accession numbers of amino acid sequences used in multiple sequence alignment and phylogenetic analysis.

Species	Member of MCRs	GenBank accession number
Danio rerio	Mc1r	AAI62836.1
	Mc2r	AAO24743.1
	Mc3r	AAI62747.1
	Mc4r	AAL85494.1
	Mc5ra	AAI62797.1
	Mc5rb	AAI63224.1
Carassius auratus	Mc1r	BAJ83471.1
	Mc2r	BAJ83472.1
	Mc3r	BAJ83473.1
	Mc4r	XP_026063043.1
Cyprinus carpio	Mc1r	AGC50885.1
	Mc2r	CAE53845.1
	Mc3r	XP_018922723.1
Xenopus tropicalis	Mc3r	XP_002935436.1
	Mc5r	AAI35996.1
Rana temporaria	Mc3r	XP_040186095.1
	Mc5r	XP_040209847.1
Chelonia mydas	MC3R	XP_007059824.1
Mus musculus	MC3R	NP_032587.1
Alligator sinensis	MC3R	XP_006018246.1
Cyanistes caeruleus	MC3R	XP_023795718.1
Gallus gallus	MC3R	BAA32555.1
Taricha granulosa	Mc3r	AAX18229.1
Pongo abelii	MC3R	PNJ37794.1
Cricetulus griseus	MC3R	XP_003501490.1
Mus Pahari	MC3R	XP_021049931.1
Sus scrofa	MC3R	NP_001116609.1
Homo sapiens	MC3R	AAH69105.1
Cyprinodon variegatus	Mc4r	XP_015237674.1
Bufo bufo	Mc5r	XP_040288553.1
Oncorhynchus tshawytscha	Mc3r	XP_024229914.1
Onychostoma macrolepis	Mc3r	MW884251
